# Prospective Comparison of Functional and Radiological Outcomes of Arthroscopic Anterior Cruciate Ligament Reconstruction by Hamstring Graft Alone and Platelet-Rich Plasma Added to the Hamstring Graft

**DOI:** 10.7759/cureus.23017

**Published:** 2022-03-10

**Authors:** Ashish Kumar, Narendra Singh Kushwaha, Deepak Kumar, Arpit Singh, Vivek Gupta, Sanjeev Kumar

**Affiliations:** 1 Orthopaedic Surgery, King George’s Medical University, Lucknow, IND; 2 Orthopaedics, King George’s Medical University, Lucknow, IND

**Keywords:** pivot-shift test, tibial tunnel, acl, lysholm knee score, lachman test, anterior drawer test

## Abstract

Aims and objectives: To measure the additional effect of platelet-rich plasma (PRP) on functional outcome of anterior cruciate ligament tear managed by augmenting anterior cruciate ligament (ACL) reconstruction with PRP.

Methods: The present study was conducted on patients with ACL tear admitted in the department of orthopaedics, King George’s Medical University, Lucknow wherein a total of 70 subjects were assigned into two groups of 35 patients each randomly, viz Group 1 in which the patients were treated by quadruple hamstring graft alone and Group 2 in which the patients were treated with augmented hamstring graft with PRP. The standardized anterior drawer test, Lachman’s test, Lysholm knee score were quantified both preoperatively and postoperatively at different follow-ups and also tibial tunnel widening was measured postoperatively at different follow-ups.

Result: The present study had 70 patients with ACL tears. The mean age of patients in non-PRP groups was 29.71 ±2.99 years while that in the PRP group was 28.34±4.32 years. On comparing the improvement in grades at pre-op, immediate postop, 6 weeks, and 3 months follow-ups, there was no statistically significant difference between the two groups. The tibial tunnel widening also showed no significant difference between the two groups.

Conclusion: In our study, it was found that both the groups showed improvements in grades of anterior drawer test and Lachman’s test postoperatively but the difference between both the groups was not significant. Similarly, while comparing the improvements in Lysholm knee score and tibial tunnel widening among both the groups, the difference was not significant. Follow up of 3 months was a limiting factor in our study. This technique needs further clinical evaluation to assess the long-term results.

## Introduction

An anterior cruciate ligament (ACL) tear is considered one of the most common injured ligament of the knee [1]. The number of patients undergoing ACL repair by tendon grafts is increasing and so is the techniques of ACL reconstruction. The ACL is a static stabilizer of anterior translation of tibia with respect to femur [2]. Origin- medial surface of lateral femoral condyle posteriorly goes distally and medially toward tibia reaching around a length of 38 mm [3]. There are two bundles of ACL- anteromedial and posterolateral-named according to their insertion on the tibia. Globally, ACL tear incidence is in the range of 36.9-60.9 per lac/year [4,5]. Also, the failure of ACL is not uncommon ranging 0.7-10% in the postop period [6,7]. Successful reconstruction of ACL requires an in-depth understanding of various factors: graft placement in normal anatomical place, mechanical properties of graft tissue, mechanical behaviour along with fixation strength of materials and the biological processes that occur during graft remodelling, maturation and incorporation [8-16]. Tendon-bone healing is expected to play an important role in the strength achieved after ACL reconstruction. Slow tendon-bone interface healing delays the rehabilitation process. So methods that can speed up and improve tendon-bone interface healing are a matter of continued research, as they can help provide early rehabilitation and prevent failures of graft function. Platelet-rich plasma (PRP), in many studies, has shown to increase the injury response and help in the process of healing [16]. Platelets are first to reach the injury site and release factors that mediate healing. From a biological aspect, animal and human in vitro and in vivo studies have shown three stages of graft healing after reconstruction of ACL: (i) early healing phase of the graft with central graft necrosis, hypocellularity and no evidence of revascularization in the graft tissue; (ii) proliferation phase, most intensive remodelling phase along with revascularization; (iii) phase of ligamentization with characteristic restructuring of graft as of an intact ACL [17-26]. At each stage, growth factors produced by platelets play a vital role, and therefore their potential function in the management of injuries to ligaments and tendons has recently come into focus. Therefore, the use of PRP to increase graft healing and ligamentization in ACL reconstruction is being studied and also whether it helps in increasing joint function and stability.

## Materials and methods

This is a prospective randomized study conducted on patients with ACL tear admitted to the department of orthopaedic surgery, of a tertiary care centre over a period of one year. Written permission from the institutional ethics committee to conduct the study was taken prior to commencement of the study. We enrolled 70 physically active patients of age group 18-45 years with clinical/radiological/arthroscopic evidence of ACL deficiency which remains symptomatic despite conservative therapy. Bilateral ACL tears, lack of fitness due to associated comorbidity, associated fractures of lower limb bones and/or spine/neurovascular injuries, associated injuries to other ligaments of the knee, significant arthritis of the knee joint and local skin infection were excluded from the study. Patients were randomized into two groups namely reconstruction with hamstring graft (Group A) and reconstruction with hamstring graft combined with augmentation with PRP (Group B) using computer-generated random number table.

All patients, who consented to be part of the study and fulfilled the inclusion criteria, were admitted to the department of orthopaedics and were assessed for age, sex, duration since the injury to intervention, anterior drawer test, Lysholm knee score, Lachman’s test and postoperative X-ray (tunnel widening) of the knee. Anterior drawer’s test, Lysholm knee score and Lachman’s test were documented preoperatively, immediate postop, 6 weeks and 12 weeks postoperatively. The patients were rehabilitated as per the standard ACL rehabilitation protocol which included gaining a range of motion, muscle strength and returning to preinjury status in phasic manner. Statistical analysis of the data generated was done using Statistical Package in the Social Sciences (SPSS, IBM Corp., Armonk, NY, United States), Microsoft excel and graph pad.

Surgical procedure protocol

All subjects were operated on under spinal anaesthesia with a tourniquet pressure of 350 mmHg with the same surgical technique of inside-out by same surgical team. We used autogenous hamstring graft in each surgery. Endo button was used at the femoral tunnel and a bio screw of diameter 1 mm more than the graft thickness was used at the tibial tunnel. In both groups, arthroscopic ACL reconstruction using autogenous hamstring graft was done in standard manner and in PRP group, the freshly prepared PRP (rapid single spin method) from the patient’s venous blood was injected in the graft and the femoral and tibial tunnels using spinal needle under arthroscopic vision at the end of the surgery.

## Results

The mean age of patients in non-PRP groups was 29.71 ±2.99 years and 28.34±4.32 years in PRP group. There were 12 females and 23 males in the non-PRP group and 13 females and 22 males in the PRP group. There was significant improvement in intragroup values for anterior drawer test, Lachman’s test and mean Lysholm knee score in both the groups with statistical significance (<0.005) but there was not much difference for these values at any follow-up when the intragroup values were compared (Table 1). In non-PRP group, preoperatively grade-3 anterior drawer test was seen in 55% and the rest of the patients had grade-2 anterior drawer test. At final follow-up, 92% patients had anterior drawer test of grade 1 or less (p-value <0.001). In PRP group, 52% patient had grade-3 anterior drawer test and the rest had grade-2 anterior drawer test preoperatively. At final follow-up, 97% patients had grade-1 or less anterior drawer test (p-value<0.001). The intergroup comparison for anterior drawer test in both groups was statistically not significant.

**Table 1:** Comparison of intra- and intergroup data for anterior drawer test, Lachman’s test and Lysholm knee score at preoperative, immediate postoperative period, at 6 weeks and at 12 weeks postoperative follow-ups and their statistical significance in terms of p-value. PRP: platelet-rich plasma; pre-op: preoperative; immediate postop: immediate postoperative; n: number

	Groups		Pre-op n (%)	Immediate postop n (%)	Postop 6 weeks n (%)	Postop 12 weeks n (%)	Intragroup comparison p-value
Lachman’s test	Non-PRP	Grade 1		33 (95%)	32 (92%)	31 (89%)	<0.001
		Grade 2	16 (45%)	2 (5%)	3 (8%)	4 (11%)	
		Grade 3	19 (55%)	-	-	-	
	PRP	Grade 1	-	34 (97%)	34 (97%)	33 (95%)	<0.001
		Grade 2	17(48%)	1 (3%)	1 (3%)	2 (5%)	
		Grade 3	18 (52%)	-	-	-	
Intergroup comparison (p-value)				0.631	0.809	0.808	
Anterior drawer test	Non-PRP	Grade 1	-	33 (95%)	32 (92%)	31 (89%)	<0.001
		Grade 2	16 (45%)	2 (5%)	3 (8%)	4 (11%)	
		Grade 3	19 (55%)	-	-	-	
	PRP	Grade 1		34 (97%)	34 (97%)	33 (95%)	<0.001
		Grade 2	17 (48%)	1 (3%)	1 (3%)	2 (5%)	
		Grade 3	18 (52%)	-	-	-	
Intergroup comparison (p-value)			0.057	0.555	0.0303	0.303	
Lysholm knee score	Non-PRP		67.67	-	80.60	90.45	<0.001
	PRP		68.48	-	80.34	90.88	<0.001
Intergroup comparison (p-value)			0.486		0.419	0.298	

Preoperatively grade-3 Lachman’s test was seen in 55% in non-PRP group and 52% in PRP group. Rest other patients had grade-2 Lachman’s test in both groups. In the non-PRP group, at final follow-up, Lachman’s test improved to grade 1 in 89 % (<0.001). While in PRP at final follow-up, Lachman’s test improved to grade 1 in approximately 95% of the cases (<0.001). The intergroup comparison for anterior drawer test in both groups was statistically not significant. Preoperatively, the mean Lysholm knee score for non-PRP group was 67.67±8.16 which improved to 90.45±3.292 at 12 weeks (<0.001) while for PRP group, preoperative value was 68.48±7.151 which improved to 90.88±3.428 <0.001) at 12 weeks. The intergroup comparison for anterior drawer test in both the group was statistically not significant. At the immediate postop, the mean tibial tunnel width for non-PRP group was 8.51±0.507, 8.79±0.503 at 6 weeks and 9.05±0.516 at 12 weeks, while for PRP group it was 8.54±0.610 at immediate postoperative period, 8.83±0.622 at 6 weeks and 9.12±0.603 at 12 weeks. There was no complication seen in any of the patients in the PRP group due to the addition of PRP to the standard ACL reconstruction procedure.

## Discussion

The study was conducted with the aim to measure the additional effect of using PRP along with hamstring graft on the functional and radiological outcome of anterior cruciate ligament reconstruction. There were no PRP related intra-op or postop complications in any subjects. In the study done by Vadalà et al. [27], all operations were performed by the same expert surgeon, they also used autogenous hamstring graft in their subjects. However, they fixed their graft at the femoral side by Swing-bridge and on the tibial side by Evolgate interference screw with a fixed diameter of 9.5 mm. In the study done by Azcárate et al. [28], all ACL reconstructions were performed by the same surgical team, using general anaesthesia. They used patellar tendon allograft for ACL reconstruction. However, they used the rigid fix technique with two biodegradable cross-pins at the femoral bone and a biodegradable interference screw at the tibial side.

In our study, we used a total of 15 ml of PRP that was injected as 3.5 ml in the tibial tunnel, 3.5 ml in femoral tunnel, 5 ml in graft substance and the remaining 3 ml intra-articularly. In the study done by Komzák et al. [29], they used 5 ml of PRP with 3 ml evenly applied on intra-articular portion of the graft and 1 ml at femoral and tibial tunnel each. In the study done by Vadalà et al. [27], 5 ml of PRP was injected at tibial tunnel and femoral tunnel each and 5 ml of PRP was applied over the intra-articular portion of the graft. In our study, we followed our patients for a period of 3 months. However In the study done by Vadalà et al. [27], patients were followed up at a median of 14.7 months (range: 10-16 months). In the study done Komzák et al., the functional status of knee was evaluated at 3 and 12 postoperative months [29]. In the study done by Azcárate et al., the follow-up of the patients was 18 months, with a mean of 24.3 months (range: 18 to 36 months) [28].

Following observations were made in our study: the mean age of patients in non-PRP groups was 29.71 ±2.99 years, which was slightly more than the PRP group where the mean age was 28.34±4.32 years. In the study done by Vadalà et al., the mean age of all 40 subjects was 34.5 years (range: 18-48 years) [27]. In the study done by Azcárate et al., non-PRP group comprised 50 patients with mean age of 26.6 years (range: 15 to 59 years) [28]. PRP group comprised 50 patients with mean age of 26.1 years (range: 14 to 57 years). There were 12 females and 23 males in the non-PRP group while the PRP group contained 13 females and 22 males. Overall, the study included 35% females and 65% males. In the study done by Vadalà et al. [27], all subjects were male. In the study done by Azcárate et al. [28], the control group of 50 patients comprised 12 female and 38 male patients. PRP group comprised 50 patients with 10 female and 40 male patients. Males constituted more subjects in both of our groups as they are more involved in outside works, sustain more injuries, road traffic accidents (RTA), etc. [30]. However in our study, the difference in gender ratio among the PRP and non-PRP was not significant (p=0.803). The mean Lysholm knee score in non-PRP group was 67.67±8.167 in the pre-op and 80.60±5.140 at 6 weeks postop and 90.45±3.292 at the 12 weeks postop. The intragroup difference in the score was highly significant. Mean Lysholm knee score in PRP group was 68.48±7.151 in the pre-op and 80.34±5.401 at 6 weeks postop and 90.88±3.428 at the 12 weeks postop. The intragroup difference in the score was highly significant. But the difference in Lysholm knee score between the two groups during the follow-up was not significant. In the study done by Vadalà et al. [27], mean Lysholm score was 55.4 ± 9.4 preoperatively and 95.6 ± 5.8 after surgical procedure in the PRP group while the mean value of the Lysholm scale raised from 57.9 ± 5.6 to 94.1 ± 3.3 in the non-PRP group. However, the difference among both the groups was not significant. Assessment of anterior drawer test at different follow-ups postoperatively showed that both the groups had improvements in the grade of anterior drawer test but no significant difference was found between the two groups.

Postoperatively in the immediate postop grade-3 was removed from both the groups. Therefore, improvement was found in both groups. Grade 2 was seen in two cases of non-PRP and one case of PRP. However, no significant difference was found between the two groups (p=0.555). At 6 weeks, grade 2 was seen in three cases of non-PRP and one case of PRP. Majority had grade 1 in both groups. However, no significant difference was found between the two groups (p=0.030). At 12 weeks, grade 2 was seen in four cases of non-PRP and two cases of PRP. Majority had grade 1 in both groups. However, no significant difference was found between the two groups (p=0.393). However, while comparing the intragroup change in the grades of anterior drawer test, it was highly significant in both non-PRP and PRP groups. In the study done by Azcárate et al. [28], tibial anterior translation in the control group was normal in 16 patients, between 5 and 7 mm in seven, and over 7 mm in two. The mean anterior displacement of the tibia in the control group was 4.5 mm (range: -2 to 9 mm). In the PRP group, tibial translation was normal in 18 patients, between 5 and 7 mm in six, and over 7 mm in one. In this group, the mean was 4.2 mm (range: -3 to 8 mm), with no statistically significant differences when compared to both groups (p>0.05).

Assessment of Lachman’s test at different follow-ups postoperatively showed that immediate postop grade 3 was eliminated from both the groups. Grade 2 was seen in two cases of non-PRP and one case of PRP. However, no significant difference was found between the two groups (p=0.555). Postoperatively at 6 weeks, grade 2 was seen in three cases of non-PRP and one case of PRP. Majority had grade 1 in both groups. However, no significant difference was found between the two groups (p=0.030). Postoperatively at 12 weeks, grade 2 was seen in four cases of non-PRP and two cases of PRP. Majority had grade 1 in both groups. However, no significant difference was found between the two groups (p=0.393). While comparing the intragroup change in the grades of Lachman’s test, it was highly significant in both non-PRP and PRP groups. In the study done by Vadalà et al. [27], all the cases in the postop of both the groups had grade-1 Lachman’s test, hence showing no significant difference.

Assessment of tibial tunnel widening at different follow-ups postoperatively showed that mean tibial tunnel width in non-PRP group was 8.51±0.507 in the immediate postop and 8.79±0.503 at 6 weeks postop and 9.05±0.516 at the 12 weeks postop. This increase in tibial tunnel width after 6 weeks was not significant. After 12 weeks, the change in width was significant (p=<0.001). Mean tibial tunnel width in PRP group was 8.54±0.610 in the immediate postop which was increased to 8.83±0.622 at 6 weeks postop. This increase in tibial tunnel width after 6 weeks was not significant (p<0.001). After 12 weeks, the mean further increased to 9.12±0.603 which was significant (p=<0.001). However, the difference in tibial tunnel width between the two groups during the follow-up was not significant. In the study done by Vadalà et al. [27], tibial tunnel diameter increased from 9.0 ± 0.2 mm to 10.9 ± 0.2 mm in PRP group (p=0.029) and from 9.0 ± 0.1 mm to 10.1 ± 0.4 mm in non-PRP, with a follow-up of 10 to 16 months. However, the difference among both the groups was not significant. In the study done by Vadalà et al.’s study [27], they also measured and compared Tegner value, International Knee Documentation Committee (IKDC) score, pivot-shift test and anterior laxity difference between the involved knee and the contralateral healthy knee but the differences among the two groups were not significant in any of them. In the study done by Komzák et al. [29], the knee functional status was evaluated at 3 and 12 postoperative months, using the scoring systems (Cincinnati score, IKDC score). The Cincinnati score at 3 months and 12 months postoperatively in the PRP group and in the control group showed no significant difference between the groups (p=0.885) in either follow-up. The IKDC score showed similar results. No complications, due to intra-articular injection/adding PRP, such as swelling, tenderness, increase in pain, redness in the joint or infection was seen in our study in PRP group patients in the postoperative period. One of the limitations of our study was the short-term follow-up period and hence the long-term effect of PRP could not be evaluated.

## Conclusions

In our study, it was found that the improvement in grades of anterior drawer test and Lachman’s test was present in both the groups postoperatively but while comparing the difference between both the groups, it was not significant, hence we can say that in our study, PRP plays no significant role in the improvement in grades of anterior drawer test and Lachman’s test. Similarly while comparing the improvement subjective Lysholm knee score among both the groups, the difference was not significant, hence PRP has no beneficiary effect in improving Lysholm knee score. The addition of PRP did not develop any complications like effusions or allergic reactions. While comparing tibial tunnel widening, the difference among two groups was not significant, hence PRP also doesn’t help in preventing tibial tunnel widening as per our study.
